# Parents' Experiences of Family‐Centred Care in Home‐Based Paediatric Care of Their Child With Life‐Limiting Illness: A Qualitative Descriptive Study

**DOI:** 10.1111/jan.16898

**Published:** 2025-03-24

**Authors:** Karjula Sari, Pölkki Tarja, Hökkä Minna, Kanste Outi

**Affiliations:** ^1^ Research Unit of Health Sciences and Technology (HST), Faculty of Medicine University of Oulu Oulu Finland; ^2^ Medical Research Center Oulu, Oulu University Hospital and University of Oulu Oulu Finland; ^3^ Diaconia University of Applied Science of Helsinki Helsinki Finland

**Keywords:** child, family‐centred care, home‐based care, life‐limiting illness, nursing, paediatric care, paediatric nursing, parents' experiences

## Abstract

**Aim:**

To describe parents' experiences of family‐centred care in home‐based paediatric care of their child with a life‐limiting illness.

**Design:**

A qualitative descriptive study with semi‐structured interviews.

**Methods:**

The purposive sample of parents (*n* = 11) of children diagnosed with life‐limiting illnesses and receiving home‐based paediatric care was recruited from a university hospital in Finland. The semi‐structured interviews were conducted between December 2020 and December 2021. The data were analysed using deductive–inductive content analysis.

**Results:**

Under the five themes of family‐centred care, 11 main categories and 31 subcategories were identified. The main categories were (1) uniqueness of all family members as care recipients, (2) incorporating family's background into care, (3) emotional support, (4) practical support, (5) information sharing, (6) negotiation, (7) parental involvement in care, (8) home care by parents, (9) collaboration with the hospital, (10) collaboration with local authorities and (11) collaboration with the home care team.

**Conclusion:**

Individually tailored interventions and carefully integrated services based on the needs of parents and all family members best support families in challenging life situations and contribute to the implementation of family‐centred care in the home‐based care of children with life‐limiting illnesses.

**Implications for the Profession and Patient Care:**

The results increase the understanding of parents' experiences, wishes and needs to support further development of a home‐based paediatric care model for children with life‐limiting illnesses.

**Impact:**

The results impact individual families caring for seriously ill children at home, professionals delivering the care and healthcare authorities and policymakers designing the services.

**Reporting Method:**

The reporting of the study is based on the Consolidated Criteria for Reporting Qualitative Research (COREQ).

**Patient and Public Contribution:**

No patient or public contribution.

**Summary**

- What does this paper contribute to the wider global clinical community?
  ○ Parents of children with life‐limiting illnesses recommend home‐based paediatric care.
  ○ Individually tailored care and services based on seamless collaboration between families, professionals and health officials best support families.
  ○ Digital opportunities are still underutilised in the home‐based care of children with life‐limiting illnesses.

## 1 Introduction

Advances in medicine and health technology have globally enabled an increasing number of children with life‐limiting illnesses to survive, be treated and live a normal life supported by a variety of medical devices and treatments and to be cared for at home (Maynard et al. 2019). A life‐limiting illness is an active, progressive or advanced disease, which may have little or no prospect of being cured, and may eventually end in death (World Health Organization, WHO 2023). To design high‐quality home‐based paediatric care (HBPC) and services for children with life‐limiting illnesses, it is crucial to understand parents' experiences and needs of care to develop positive collaborative family‐centred care for families and children with life‐limiting illnesses (Institute for Patient‐ and Family‐Centered Care, IPFCC 2024; Winger et al. 2020).

## 2 Background

Families and children often experience the impact of the hospital environment as negative for their well‐being and the family's everyday life. Whenever possible, they choose home‐based care over hospital care because it can be tailored to the needs of child's illness and the family's resources. (Hansson et al. 2023). HBPC has been shown to increase both children's and parents' satisfaction with care and to improve their quality of life (Effendy et al. 2022; Winger et al. 2020). International studies show that HBPC remains underprovided, particularly for children with life‐limiting illnesses and children with complex medical needs (Maynard et al. 2019). There is no justification for unnecessary hospitalisation and prolonging the discharge of children, as each child is part of their family and belongs at home whenever possible (European Association of Children in Hospital, EACH 2022).

Family‐centred care (FCC), based on the United Nations Convention on the Rights of the Child (United Nations, UN 1989), states the rights of children and families to participate in the planning, implementation and evaluation of their own healthcare. A family‐centred approach is characterised by recognition and respect for the uniqueness and individuality of each family, holistic consideration and support for all family members, parents' voluntary participation at their level of comfort, open communication and mutually beneficial collaboration between the family and healthcare professionals in the planning and decision‐making based on the family's needs, opinions and wishes (Institute for Patient‐ and Family‐Centered Care, IPFCC 2024; O'Connor et al. 2019; Smith 2018).

FCC has a long tradition in paediatric healthcare, but the approach is also suitable for the whole healthcare context (Kokorelias et al. 2019) as in FCC; the patient and family are seen as essential partners in the care of people of all ages (O'Connor et al. 2019). The patients and families themselves define their family and decide how they will be involved in care and decision‐making (Institute for Patient‐ and Family‐Centered Care, IPFCC 2024). The family is seen as an integrated unit, and support is offered to all family members (Smith 2018). FCC has been shown to promote the health and well‐being of individuals and families, to improve the quality of care and services (Seniwati et al. 2023), the family's experience of care (Arabiat et al. 2018) and the job satisfaction of care staff (Park et al. 2018) and to facilitate the appropriate allocation of healthcare resources (Seniwati et al. 2023). FCC is an approach that recognises the crucial role of the family in the patient's life and encourages mutually beneficial collaboration between patients, families and healthcare professionals (Hammer et al. 2023; Toivonen et al. 2020).

FCC has been studied quite extensively in inpatient paediatric hospital settings (Abukari and Schmollgruber 2023), identifying the need for improvement in including parents in decision‐making and communication (Terp et al. 2021). In addition, it has been concluded that more education should be provided for nurses to ensure the implementation of FCC practices in paediatric inpatient clinics (Cetintas et al. 2023; Dall'Oglio et al. 2018). However, there is still a paucity of research on FCC in the HBPC context. HBPC and services at their best not only support children's rights but also the realisation of FCC (Effendy et al. 2022; Thienprayoon et al. 2020).

## 3 The Study

### 3.1 Aim and Research Question

The aim of this study was to describe parents' experiences of family‐centred care in the home‐based paediatric care of their child with a life‐limiting illness. The research question was: What kind of experiences do parents have of family‐centred care in the home‐based paediatric care of their child with a life‐limiting illness?

## 4 Methods

### 4.1 Design

A qualitative descriptive study design with semi‐structured interviews (Polit and Beck 2021) was used because the purpose of the study was to increase the understanding of parents' experiences of FCC, as well as their wishes and needs when caring for their child with a life‐limiting illness at home and to provide information to support the development of HBPC. The study followed the Consolidated Criteria for Reporting Qualitative Studies (COREQ) (Tong et al. 2007) guidelines (Table S1).

### 4.2 Theoretical Framework

FCC provided the theoretical framework for the study (Smith 2018). The data collected in interviews were approached first deductively according to the characteristics of FCC and then inductively (Kyngäs et al. 2020) to gain a deeper understanding of parents' experiences of FCC in HBPC.

### 4.3 Setting and Recruitment

A purposive sampling technique was used to recruit parents who were interviewed (Polit and Beck 2021) in a paediatric clinic of a university hospital in Finland. An information letter with a request to carry out the study was sent by the first author to the ward manager of three departments of the hospital in question. The ward manager identified potential parents of children diagnosed with different life‐limiting illnesses to obtain a sample that was representative in HBPC at the hospital. These parents were then contacted by the first author by phone, and thereafter they were sent an invitation letter by e‐mail containing information about the study.

### 4.4 Participants

There were 11 participants involved in the research: nine women and two men. The children of the participants had initially been treated in three university hospitals in Finland but have since been discharged to HBPC, which has been implemented in different ways for each child and family, depending on the child's illness and the parents' wishes. Three families had a 24‐h home care service organised by a public or private service provider, while others had a nurse from the hospital or from a home care service provider visiting the family during the treatments or as needed. One family preferred to take care of the treatments all by themselves.

Parents whose children under the age of 18 years had been diagnosed with life‐limiting illnesses such as cancer or severe congenital diseases and had been in HBPC were included in the study. Most of the children had palliative care needs, and three of the participants were bereaved parents. Four of the children were girls, and seven of them were boys. The children were aged between 5 months and 16 years (average 6 years). The time spent caring for the child at home ranged from 2 weeks to 11 years.

### 4.5 Data Collection

The participants were interviewed using a semi‐structured interview with themes deduced from the characteristics of FCC (Table 1). A pre‐interview was conducted with one participant, and no changes were applied to the interview guide. Individual face‐to‐face interviews were conducted by the first author (SK) between December 2020 and December 2021, at a time and location chosen by the participants: the first two on the hospital premises and the remaining nine remotely. In the case of remote participation, the parents were allowed to keep the camera on if they wished. At the beginning of each interview, the researcher explained the aim of the study and data collection techniques. All interviews were conducted in Finnish, with an average length of 78 min (range 39–118 min), the shortest interview being given by a bereaved parent who insisted on participating regardless of the sensitivity of the research topic. The interviews were digitally audio‐recorded by a computer speech recorder and transcribed verbatim, resulting in a 146‐page written research Word document (Times New Roman 12, line spacing 1.5). Data saturation was achieved by the eighth interview when no additional codes were identified, and a further three interviews were conducted to confirm saturation of the data. No field notes were taken.

**TABLE 1:** The interview guide.

1. Respect for uniqueness of family How do you experience the individuality of your family has been considered? How have all family members been taken into account? How has home‐based care of your child affected your family?
2. Support provided to family How do you experience being supported during the home‐based care of your child? What kind of support have you and all your family members received? What kind of support would have been useful?
3. Communication How do you experience the communication between home, professionals and local authorities? What kind of experiences do you have about the information sharing with professionals? How do you experience about being included in decision‐making concerning your child? How do you communicate with professionals? (visits, discussions, phone calls, digitals access)
4. Parental partnership How do you experience of being involved in your child's care and treatments in home‐based care? How do you experience your expertise is being respected concerning your child's care?
5. Collaboration How do you experience the collaboration between home, professionals and local authorities? How do experience the competence of the professionals included in your child's home‐based care?

### 4.6 Data Analysis

The data analysis was conducted by the first author (SK) using a deductive‐inductive content analysis (Kyngäs et al. 2020). The analysis matrix consisted of five themes: respect for the uniqueness of family, support provided to family, communication, parental partnership and collaboration, that were derived from the concept analyses of FCC (IPFCC, 2024; O'Connor et al. 2019; Smith 2018). The audio‐recorded interviews were transcribed verbatim, the transcripts were compared to the recordings and carefully read through several times to obtain an overall picture of the data. A sentence was chosen as the unit of analysis, and authentic expressions were simplified. The simplified expressions of the data were first classified deductively using the five themes of FCC in the analysis matrix, and then the data were analysed inductively under each theme, forming subcategories and main categories to answer the research question. An example of inductive content analysis process under the theme of Respect for uniqueness of family is demonstrated in Table 2. The analysis was conducted on Finnish data and the final analysis, with the authentic quotations, was translated into English.

**TABLE 2:** Example of the inductive content analysis process within the theme of respect for the uniqueness of family.

Simplified expressions	Subcategories	Main categories
Possibility to be present and close to the child at home Normal family life at home Child longing for his own belongings at home Child missing his/her siblings Family wish to adapt child's medication to family life Child prefers to be at home Child is an equal member of the family at home Family members need to be together Good for siblings to have parents at home Normal relationship between siblings at home	Unity of family in everyday life	Uniqueness of all family members as care recipients
Service voucher model not flexible Family was not asked what kind of services they needed No support for family coping Wish to have some help in cleaning Need of domestic help No real help what the family would have needed Tiring to have too many people trotting in and out at home More tailor made services for family More individual services Responding to family's individual needs No help to be offered for an exhausted mother	Lack of individuality	
Sibling got anxious about the strangers in the house Sibling said he hated the nurses Not enough resources for the siblings Siblings feel left out Everybody is concerned about the sick child Sibling grew up as an invisible kid Siblings worry about the dying child Sibling takes too much responsibility of household duties Siblings worry about parents' well‐being	Insufficient attention to siblings	
Child's desire to get up and out until the end was honoured Nurses enabled everything the family wanted Raising up the child parents' responsibility Respecting parents' opinions Collecting the child's body an unpleasant experience	Honouring family's choices	Incorporating family's background in the care
Strangers in the house limited family life Nurses' presence at home disturbed siblings Marital relationship was limited Family's social life was limited Sleep of family members was disturbed	Loosing privacy of family and home	
Cost of care made parents feel guilty Expensive medication made parents feel guilty Local officers blamed parents for not caring by themselves Asking help made parents feel low Parents blame themselves for the situation Parents feel guilty for not being able to pay attention to siblings	Feeling guilty	

### 4.7 Ethical Considerations

This study was conducted in accordance with the Finnish National Board on Research Integrity (Finnish National Board on Research Integrity TENK 2023) and did not require formal approval from an ethics committee because it did not interfere with the physical or mental integrity of human beings, it was not a study on the causes, symptoms, diagnosis, treatment or prevention of diseases and the participants were not under 18 years old. The study permission (277/2020) was obtained from the university hospital. Participation in the study was voluntary, and the participants had the option to discontinue their participation at any time. The participants were informed about the aim of the study, the duration and recording of the interview, the contact details of the researcher and that the discussions would be confidential. The measures required by the General Data Protection Regulation (General data protection regulation, GDPR 2016/679) were carefully considered throughout the study. All participants signed their informed consent prior to data collection, and anonymity was assured by assigning an identification number to each participant.

### 4.8 Trustworthiness

The trustworthiness of this study has been judged according to Lincoln and Guba (1985) by assessing credibility, dependability, confirmability, authenticity and transferability (Polit and Beck 2021). Careful design, a detailed description of the analysis, systematically reported results and the diverse backgrounds of the research team (Table S2) strengthen the credibility of the study. To enhance dependability, the first author conducted all the interviews according to exactly the same interview guide. The confirmability of the study was strengthened by employing reflexivity for transparency and consistency throughout the analysis process. To enhance authenticity, the participants' anonymised quotations have been presented to support the study findings. The children of the participants had been inpatients of three different university hospitals with different practices regarding FCC, which strengthened the transferability of the results. The research results have been reported as accurately as possible, so readers are able to decide on the transferability of the results to a specific context. The trustworthiness of the study was also enhanced by saturation of the data (Kyngäs et al. 2020).

The relationships between the researchers as well as the aspects of conducting the study were clearly indicated. Regular discussions with the research team also reduce researcher bias during the analysis. No relationship between the participants and the researcher existed previously.

## 5 Findings

The findings highlight the gratefulness of parents for the availability of HBPC for their child with life‐limiting illness. All participants recommended HBPC, even though it was burdensome and often strained the resources of the family. They requested access to services that would be tailored to the family's needs because excessive services and unnecessary strangers in the family home also increase the burden on an already challenging situation.

The parents' experiences of FCC in HBPC are described under five themes: experiences of respect for uniqueness of family, support provided to family, communication, parental partnership and collaboration (Table 3). In total, 11 main categories and 31 subcategories were identified from the data. The main categories were (1) uniqueness of all family members as care recipients, (2) incorporating family's background into care, (3) emotional support, (4) practical support, (5) information sharing, (6) negotiation, (7) parental involvement in care, (8) home care by parents, (9) collaboration with the hospital, (10) collaboration with local officials and (11) collaboration with the home care team.

**TABLE 3:** Parents’ experiences of family‐centred care in home‐based paediatric care of their child with a life‐limiting illness.

Respect for uniqueness of family	Support provided to family	Communication	Parental partnership	Collaboration
**Uniqueness of all family members as care recipients** Unity of family in everyday life Lack of individuality Insufficient attention to siblings	**Emotional support** Creating sense of safety and trust Providing conversational help to all family members Providing peer support	**Information sharing** Encouraging atmosphere Providing information Lack of digital communication	**Parental involvement in care** Parental participation at their level of comfort Parental responsibility for introduction of home care team Parental involvement in care during child's hospital stay	**Collaboration with hospital** Enabling home care Providing direct connection to hospital Acute situations challenge parents
**Incorporating family's background into care** Honouring family's choices Losing privacy of family and home Feeling guilty	**Practical support** Organising facilities for home care Financial support Supportive services provided to family	**Negotiation** Appreciation of parents' expertise Inclusion of parents in decision‐making	**Home care‐by‐parents** Parental deliberate total responsibility for home care Overwhelming responsibility for home care for parents Effects of caring responsibility on parenting	**Collaboration with local officials** Fighting for statutory benefits and services Ignoring medical statements and instructions
				**Collaboration with the home care team** Home care team as a resource Lack of competence within the home care team Insufficient deputy system

### 5.1 Respect for Uniqueness of Family

The parents' experiences of respect for the uniqueness of the family included two main categories: (1) uniqueness of all family members as care recipients and (2) incorporating family's background in care.

#### 5.1.1 Uniqueness of All Family Members as Care Recipients

The main category of the uniqueness of all family members as care recipients included three subcategories: (1) unity of family in everyday life, (2) lack of individuality and (3) insufficient attention to siblings.

The parents described the possibility of having their child in home care as extremely important for the formation and maintenance of normal relationships between family members and siblings in everyday life. According to the parents, the children themselves also expressed their wish to be reunited with the family and go home to a familiar environment. The parents felt that mostly they had been considered and helped as a whole family when planning and implementing care arrangements, though in some situations they would have appreciated more flexibility with the child's treatments to allow the child to experience family unity.

We were allowed to be present and close to him (during terminal care) as much as each of us wanted. [Interview 3]

Concerning individuality, the parents reported that the family had been considered in the planning and implementation of services but had not been specifically asked what kind of help they would have needed. They experienced that they did not receive individually designed services but were offered ready‐made packages of services. Some parents expressed their concern that families with 24‐h home care service are not eligible for any other help or services. The parents felt that round‐the‐clock home care is not only a resource but also a burdening factor for the family.

It was sometimes quite tiring to have people trotting in and out that we are now helping, but it was not the real help we would have needed. [Interview 7]

The parents shared their concerns about insufficient attention given to the siblings. According to the parents' experience the siblings often feel left out when the main attention of parents and carers is focused on the child with a severe condition, and this is reflected as anxiety in the sibling's behaviour. The parents expressed the wish that there would be someone outside the family who would pay special attention to the well‐being of the siblings because, as parents, they did not always have the resources for this in a difficult situation.

He (brother) remembered his life before the sister's homecare, and he talked about it many times, how he hated the nurses. [Interview 5]

#### 5.1.2 Incorporating Family's Background Into Care

This main category of incorporating the family's background into the care included three subcategories: (1) honouring family's choices, (2) losing privacy of family and home and (3) feeling guilty.

The participating parents felt that their family background, values and choices were generally honoured and respected in the planning and implementation of their child's care. As the nursing staff got to know the child and the family better, their understanding and respect for the family and their choices strengthened. Unfortunately, most parents (*n* = 8) had experienced situations where their choices had been criticised or were not valued, or their wishes and opinions had been ignored in the discussions with staff regarding the child's upbringing or continuing care. Sadly, the process of retrieving the child's body after a home death had been beautifully described for the family in advance, but it had been the most unpleasant experience of the entire period of the child's end‐of‐life home care.

Then there came two rough guys who slammed a stretcher and a plastic tarp on the floor, and to them, the child's body should have been given. [Interview 3]

All parents shared that losing the privacy of their family and home because of their child in home care was a disappointing surprise and that they found it more burdensome than they could have imagined. Families' expectations of a wonderful homecoming after a long and tiring hospital stay were replaced by the constant presence of a stranger in their own house, which was emotionally distressing for all family members. According to the parents' experience, the continuous presence of nursing staff at home and the sounds of medical equipment and treatments disturbed not only the sleep of the family members, but also limited normal family life, the marital relationship, and the family's other social life. Some parents reported that they had had to arrange a separate room, or even move to a larger house, not only to provide space for the child's home care but also to ensure some privacy for the rest of the family, which unequalised families based on their economic situation.

I had in my mind all the time that you couldn't argue, and everything else that is in a marital relationship had to be so that the nurse couldn't hear. Yes, it was limiting. [Interview 8]

A few parents expressed a sense of guilt about their own situation and their need for help in the family when dealing with local social and health administration officials, even though these are statutory services and supports that the family is entitled to. In an already difficult situation, the parents of a seriously ill child felt that justifying their need for help was unacceptable and increased the burden on their family.

We were told that don't you realize that you are the most expensive family in the county anyway? [Interview 4]

### 5.2 Support Provided to Family

The parents' experiences of support provided to the family included two main categories: (1) emotional support and (2) practical support.

#### 5.2.1 Emotional Support

This main category of emotional support consisted of three subcategories: (1) creating a sense of safety and trust, (2) providing conversational help to all family members and (3) providing peer support.

The parents described how a sense of safety and trust is crucial in supporting parental coping while caring for their seriously ill child at home. They named the competence of the home care team, the intensive contact with the caring unit and the knowledge of the possibility of being re‐admitted to the ward as factors which increased the feeling of safety at home. The discharge from hospital to home care or changing the home care team from one provider to another was the most critical situation in terms of feeling safe. The parents trusted the nurses in the ward more than the nurses in the home care team, regardless of the service provider being private or public. The parents would have preferred the nurses from the ward to visit them at home to deliver the most demanding care and treatments. The concrete presence of caregivers was considered very important by the parents and increased their sense of safety in difficult circumstances.

It was like a very stressful situation for me as a mother when I couldn't trust everyone's professionalism and expertise, so I didn't feel safe. [Interview 5]

The parents reported that there was quite a good access to psychological counselling for the child and parents, but also for siblings, on behalf of the university hospital, when the child was being treated in the ward, whereas during home care, the availability of counselling was poor. However, during bereavement of the child, families had had access to counselling, which they had found to be an important support in the grieving process. Moreover, the parents felt that conversations not only with the psychologist and social worker but also with nurses and doctors were important. However, the parents wished for more continuity and flexibility in the hospital's psychology services and they preferred to have discussions in the child's patient room in a safe environment rather than in the psychologist's room away from the ward. According to the parents, during home care, telephone contacts with the psychologist also appeared to work well for parents and older children. Overall, the parents expressed the wish that more attention should be paid to their well‐being, although the focus is on the care of the child's serious illness, as parents never decline to take care of their child, even if they are already tired.

As a parent, you just do not think early enough to say that I'm not up to it anymore. So, in a way at the stage when the parent says that I'm not doing so well, it is already a pretty bad situation. [Interview 7]

The parents valued peer support whenever it had been available. Some families had been able to attend peer family meetings organised by the hospital as soon as the diagnosis of the child's illness was confirmed, which they had found to be of great importance to all family members. The contacts between peer families had continued for years, sharing both practical tips for coping with everyday life and feelings about the burdensome situation with a severely ill child. The parents also mentioned that they had received peer support through social media peer support groups and various courses offered by patient organisations. Some parents had also informed the hospital that they would be available if their experiences could be of help and benefit to other families.

As soon as the doctor told us what kind of illness this was, he immediately mentioned families from the area, and we met them there in the hospital, which was wonderful in the very early stages. [Interview 6]

#### 5.2.2 Practical Support

This main category of practical support consisted of three subcategories: (1) organising facilities for home care, (2) financial support and (3) supportive services provided to families.

Most parents reported that the initial idea of considering home‐based care came from the staff of the department, as the parents themselves had no knowledge of such a possibility nor the resources to even consider different options. A few parents said that the child had started to ask about going home, but they also initiated it themselves because it was overwhelming for the family to be in hospital with a seriously ill child at the same time as having siblings at home, especially those families who lived quite a distance away. The parents were very satisfied with the staff in the department who organised the child's home care by instructing the local health authorities on preparations for the child's home care, such as the necessary facilities, competent staff, and medical equipment and medication.

The child in such pain that any possible movement hurt, so they tried to bring equipment such as different carrying and lifting cloths, for example, which could help. [Interview 3]

The parents reported that the financial support provided by society had been crucial and enabled them to take time off work to care for their child at home when necessary. Caring for a child with a life‐limiting illness is a major burden on the family itself, and therefore the parents felt that the family care allowance and other financial benefits, such as the various commitments to pay for medicines and medical supplies, have significantly helped the family.

Dad has also been at home full‐time with a special care allowance and economic support for family care, and that's important. [Interview 6]

The participating parents indicated that the appropriate supporting services provided by society significantly enhanced the ability of families to cope with the challenges of their child's illness and treatment, providing that these services were individually tailored to the family's unique needs. The parents expressed a wish for concrete domestic help in everyday life at home, such as help with cleaning and looking after the siblings, as well as the possibility of respite care for the child. The parents also expressed their concern that if the family is offered a round‐the‐clock home care service for the child, the family will lose not only the family carer's allowance, but all the supporting services associated with this allowance, such as the statutory family carer's monthly leave. According to the parents, there was very little or no support from the child's health clinic to access appropriate family services to support family coping.

There should be some help to care for the other children at home sometimes so that the parents can have a break sometimes. [Interview 8]

### 5.3 Communication

The parents' experiences of communication included two main categories: (1) information sharing and (2) negotiation.

#### 5.3.1 Information Sharing

This main category of information sharing consisted of three subcategories: (1) encouraging atmosphere, (2) providing information and (3) lack of digital communication.

When asked about the experience of communication with nursing and medical staff, the parents reported that they had found the interaction and atmosphere to be very straightforward and supportive, encouraging the parents to ask questions and contact both the home care team and the staff on the ward. According to the parents' experiences, the nursing staff had been positive in their response and had encouraged the parents and family in a difficult period.

They are very encouraging; it's been awesome, and they give praise and so much positive feedback. [Interview 2]

The parents felt that they had been well informed by the staff of the department about their child's illness and treatment. They also reported having received very specific instructions on how to deal with the child's end‐of‐life care, possible home death, and other acute situations. However, the parents would have preferred to have been better informed about the timing of the child's discharge and the provider of the home care service. According to the parents, the information about the changes of home care provider from one to another had been concealed. In addition, a few parents reported that access to information about possible forms of support, both financial and social, was variable, and families felt that they had to find out by themselves what kind of help and support they would be entitled to.

It's not fair that when the parents are tired in the middle of it all, they must think about what kind of support and help they should be getting now. [Interview 7]

Communication between the family and professionals occurred mainly through face‐to‐face home or hospital visits. Families were allowed to contact the ward by telephone, which they considered an important safety factor. One family reported a very facilitating practice where the doctor called about the treatment instructions based on blood test results, and the family did not have to travel to the hospital. Apart from telephone calls, families had no other forms of digital communication, such as video consultations with the hospital team or remote medical monitoring devices.

There should have been check‐up calls from the ward on a certain day of the week at a certain time to ask how things were going; it would have given a sense of security. [Interview 7]

#### 5.3.2 Negotiation

This main category of negotiation consisted of two subcategories: (1) appreciation of parents' expertise and (2) inclusion of parents in decision‐making.

The parents reported that their expertise had been appreciated by the nursing and medical staff in relation to their child's illness and treatment, which had been very meaningful to them as they had worked so much for the child and felt they were the best experts concerning the child's situation. The parents felt that the trust of the hospital staff in their expertise had empowered them and that they felt they could cope better at home when they were trusted. Nevertheless, a few parents reported that they felt that their expertise had been ignored, especially in emergency situations, as their concerns about their child's state had not been taken seriously enough, resulting in further weakening of the child's condition.

When a parent says that now there is something wrong so it is completely true and that should be considered seriously. [Interview 7]

The participating parents reported that they felt that they, as parents, were well considered as the main partner in the decision‐making about the child's care, regardless of whether there was a 24‐h home care team available or not. However, they had felt excluded from the process of planning the child's home care arrangements and recruiting the home care team, even though caring for the child in the family home, whatever the arrangements, always has a significant impact on the family's daily life.

We were not consulted or asked in any way when they were recruiting nurses for the home nursing team. [Interview 11]

### 5.4 Parental Partnership

The parents' experiences of parental partnership included two main categories: (1) parental involvement in care and (2) homecare‐by‐parents.

#### 5.4.1 Parental Involvement in Care

This main category of parental involvement in the care consisted of three subcategories: (1) parental participation at their level of comfort, (2) parental responsibility for the introduction of the home care team and (3) parental involvement in care during the child's hospital stay.

In most of the families in the study, the care of the child at home was mainly the responsibility of the home care team, and parents participated in the care at their level of comfort, according to their own resources, either by taking shifts as a member of the care team or just by being involved as a parent. Some parents reported that they would have preferred to work as part of the home care team so that the family could have more intimate family time without having a stranger in the house all the time, but this was not allowed either because of the child's illness or the local officials.

I ended up as one of the caregivers, so we were able to be as a family at home every week on certain evenings and days without having a stranger at home. [Interview 8]

The parents reported that they, as parents, were mainly responsible for training the nurses in the home care team, as even the qualified nurses needed a lot of parental support and guidance at the beginning when caring for the child. In addition, the parents experienced a sense of responsibility for their child's care despite the 24‐h presence of the home care team, as nurses nevertheless relied on the parents regarding various aspects of their child's care.

It was very difficult at the beginning as nurses needed a lot of parents' help with the care. [Interview 5]

The parents reported various experiences of parental involvement in the child's care in different hospitals with one hospital expecting them as parents to take care of the child from morning to night, while in another hospital parents were encouraged to take time off during the child's hospital stay as parents have a great responsibility for the care at home. Some parents even reported that they had had to go to hospital to perform certain treatments for the child because the nursing staff on the ward did not have the required competence.

Sometimes we have had to go to hospital ourselves because there has not been a nurse there who knew how to change a nasogastric tube or do the dialysis treatment. [Interview 6]

#### 5.4.2 Homecare by Parents

This main category of homecare‐by‐parents consisted of three subcategories: (1) parental deliberate total responsibility for homecare, (2) overwhelming responsibility for homecare for parents and (3) effects of caring responsibility on parenting.

Some of the parents expressed their great appreciation for the opportunity to care for their child by themselves at home, supported by the hospital, which had been a good arrangement for both the child and the family as it enabled the family to continue everyday life as normally as possible without the constant presence of strangers in the house. These parents reported that they had been very enthusiastic about learning all the procedures involved in caring for the child to be able to manage the care at home as much as possible, because at home the child also felt better both mentally and physically, was more active and, in their experience, was better protected from infections.

In any case, we didn't want a home care nurse because we were taking care of the child in our bedroom, and we didn't want a nurse to come here at night. [Interview 6]

While some parents were satisfied with the possibility of caring for their child at home entirely by themselves, other parents considered it too overwhelming without the help of a home care team. These parents felt that at the very least, a home care nurse should be present during demanding medical treatments and medication, or there should be the possibility for the family to have access to the ward for the treatment of their child. Some parents would have preferred to be able to take their child to a nearby hospital for overnight treatments, but this was not possible because the hospital had no competent staff, even though the parents had learned to perform the same medical procedures at home.

It was just surviving, and it unfortunately affects the marriage and family life and everything; it's been too demanding, with not enough support provided at home. [Interview 7]

Some of the overwhelmed parents reported that the constant and excessive home caring responsibility and necessity to perform difficult and even painful procedures on their own child had a negative emotional effect on their parenthood and the child–parent relationship, to the extent that they felt they were unable to enjoy parenting at the most difficult times because the time spent at home was full of preparing and performing the treatments as well as worrying about the child's condition.

Sometimes I felt like I wasn't even a mother to the child when it was kind of caring for her all the time. [Interview 7]

### 5.5 Collaboration

Parents' experiences of collaboration included three main categories: (1) collaboration with hospitals, (2) collaboration with local officials and (3) collaboration with home nursing teams.

#### 5.5.1 Collaboration With the Hospital

This main category of collaboration with hospital consisted of three subcategories: (1) enabling home care, (2) providing direct connection to hospital and (3) acute situations challenging the parents.

The parents considered collaboration with the university hospital staff to be crucial for the successful care of their child at home, as the department staff were proactive and had a central enabling role in organising the home care services in co‐operation with local officials and service providers. The parents preferred to rely on the staff of the department rather than the home care team because the department staff had the best understanding of the child's illness and its treatment, as well as the family's resources and ability to care for the child at home. Unfortunately, despite the hospital's careful prior arrangements, there were often unexpected challenges in the transition to home care, particularly in terms of the competence and the number of nursing staff provided by the home care service provider.

The hospital staff has always thought a lot about the family and what is best for us because it's a long journey to the hospital and we also had other children, so it wasn't always so easy to get around the hospital. [Interview 6]

The parents also reported that a direct connection to the university hospital and the ward staff was very important, regardless of whether there was a 24‐h home care service available. The telephone contacts with doctors and nurses in hospital, as well as permission to go directly to the ward if necessary, not only enhanced the family's coping but also reduced unnecessary hospital visits as parents and home care team were instructed by phone on child's care according to the child's current condition and the laboratory results.

We have been given permission always go to the ward directly, which is always such a big and relieving thing. [Interview 4]

According to some parents, the most challenging situations occur during on‐call hours in the hospital emergency when the doctors treating the child are not available and the parents' concerns about their child's state are not considered seriously enough, resulting in some cases in a further weakening of the child's condition.

As a parent, you must put yourself in a very unpleasant role so that you have to threaten and shout to get things to happen if it looks like the child is going to die. [Interview 7]

#### 5.5.2 Collaboration With Local Officials

This main category of collaboration with local officials consisted of two subcategories: (1) fighting for statutory benefits and services and (2) ignoring medical statements and instructions.

The parents expressed their gratitude for the care and services they have received but complained that they have had to constantly fight with local social and health officials even for statutory benefits and services that they should automatically be entitled to, which consumes their already strained resources. According to the parents, there are varying policies in different parts of the country, and public officers seem to have varying interpretations of existing laws and regulations, which leaves families in an unequal position depending on the area where they live. They also expressed the view that the best interests of the family and the child should always be considered because the illness and care of the child place enough strain on the family in themselves, and all positive help and support is needed to help the family.

All the time there are battles going on with the local authorities, which is a shame, and all this help and support we have received when we have been pretty tough at this. [Interview 7]

A few parents reported being very offended that the local officials had ignored the medical statements and instructions about the child's care and need for services at home, without seeing the child or contacting the family or the doctor. They expressed their concern that ignoring the medical advice might result in hospitalisation of the child.

When a child with special needs is born into a family, which is already a big deal for the family and it is quite a burden, so in such a situation you would hope that the burden would not come from elsewhere. [Interview 4]

#### 5.5.3 Collaboration With the Home Care Team

This main category of collaboration with the home care team consisted of three subcategories: (1) home care team as a resource, (2) lack of competence within the home care team and (3) insufficient deputy system.

The parents reported that the home care teams proved to be a great source of strength for the family, as the parents' trust in the nurses' professional competence grew and the family and the nurses learned to know each other better, although initially all family members were stressed by the presence of strangers in their own homes.

The nurses have been the biggest resource in helping us to cope. [Interview 5]

According to the parents, the professional competence of the home care team was sometimes insufficient for the specific care of the child, leaving the parents to take responsibility for both caring for the child and providing guidance to the nurses. In addition, the parents reported that despite careful arrangements, there were often unexpected challenges in the transition to home care or from one home care service provider to another, particularly in terms of the competence and sufficient number of nursing staff within the home care team.

If you have a tracheostomy patient, every nurse should know what to do if a tracheostoma comes loose, and as a mother, I don't have to worry about taking responsibility or teaching a nurse in a situation like this. [Interview 5]

The parents reported that the home care service providers could not always guarantee 24‐h service, although the family was entitled to it because they did not have a reliable substitute system for the home care team. In case of illness, one of the parents had to cover for the nurses, or there was a nurse on shift who did not have the required competence in the specific care of the child, which burdened the family constantly and complicated the parents' employment.

We should not have such a burden on our shoulders that when this homecare system collapses. [Interview 11]

## 6 Discussion

This study produced novel knowledge about FCC in HBPC of children with life‐limiting illnesses, describing parents' experiences on five themes related to FCC: respect for uniqueness of family, support provided to family, communication, parental partnership and collaboration. The results revealed that the parents had variable experiences of FCC, for example, in terms of identifying the individual resources of the family when planning and delivering the child's home care and necessary supporting services in collaboration with local authorities. The parents shared the experience of the crucial importance of the holistic family‐centred support provided by the university hospital team when caring for their seriously ill child at home.

The results of this study identified the lack of individuality and losing privacy as the key experiences of parents in respecting the uniqueness of the family in the home‐based care of a child with a life‐limiting illness. Losing privacy was one of the most difficult issues for both parents and siblings during the child's home‐based care. The findings emphasise not only the importance of identifying the individual resources and needs of families, but also the impact of a child's life‐limiting illness on all family members, making it crucial to consider not only the child and parents but also siblings in the home‐based care of the child. Our findings further highlight that the same interventions and services are not appropriate for every family, as some families are able to cope with very little help and support in everyday life in a challenging situation, while others need much more support and services to be able to manage at home with a seriously ill child, which is also supported by Winger et al. (2020). A better understanding of the unique needs and experiences of the parents of a child with a life‐limiting illness is a key factor in the development of positive collaborative care and services for families (Hammer et al. 2023). Our findings also strongly underline the importance of family unity and the role of the family in the life of the child despite a life‐limiting illness, which is also emphasised widely in previous studies (Seniwati et al. 2023; Effendy et al. 2022), as home‐based care allows family members to spend time together in daily life as much as they prefer.

Our findings regarding the support provided to the family underlined the importance of competent nursing staff in creating a sense of safety for the family in the home‐based care of a child with a life‐limiting illness, as experiencing a sense of safety improved the family's ability to cope with caring for the child at home. The support provided by society and the healthcare system was crucial for the families, as without that support, the child would have spent the rest of the life in institutional care. To provide appropriate and tailored support, professionals need to understand parents' perceptions, relationships with their child and coping strategies. Individually tailored support provided at home strengthens the family, improves their quality of life and reduces unnecessary hospital visits (Effendy et al. 2022).

The results in terms of communication highlight the parents' empowerment and experience of being heard in matters related to the child's care, with the exception of the recruitment of a home care team, discharging the child from hospital or switching the home care provider to another, where parents felt excluded, even though the findings of Thienprayoon et al. (2020) indicate that a competent home care team is crucial for the daily life of the child and the family. Our findings further reveal that communication between the family and professionals still relied mainly on face‐to‐face contacts or telephone calls, and no other forms of digital services, such as video visits or consultations with the hospital team, nor remote monitoring or transmitting of recorded information were used, although advanced digital methods would facilitate the provision of family‐centred services independent of the time and place, also for children with life‐limiting illnesses in home‐based care.

According to the results, parents of children with life‐limiting illnesses are determined to be involved in the care of their child at home and to be educated in all the necessary medical and nursing procedures before the child's discharge. However, it is important to understand that FCC is not only about involving parents in the care of their child but also about allowing parents to participate on their level of comfort and within the limits of their own resources, which is also supported by Winger et al. (2020). Our results raised the concern that to be able to stay in home care with the child as much as possible, parents sometimes have to undertake such demanding medical procedures that not even all professionals are able to perform without additional training.

According to our findings, the collaboration with both hospital staff and the home care team was considered beneficial and open, but in many cases, the collaboration with local health and social officials was often inflexible and made everyday life even more difficult for the family. As FCC is defined as an approach that encourages mutually beneficial collaboration between families and healthcare professionals (Hammer et al. 2023; Toivonen et al. 2020), our findings further highlight the need for individually tailored care and integrated health and social services for families of children with life‐limiting illnesses in HBPC.

### 6.1 Limitations

This is one of the first studies on the family‐centred HBPC of children with life‐limiting illnesses; however, some limitations must be acknowledged while interpreting the results. The sample of participants in the study was relatively small, although saturation was ensured while collecting the data. Furthermore, the transcripts were not returned to participants before analysis, but in all phases of the analysis, the transcription was compared to the audio‐recorded data. In addition, the first author (SK) was aware that her own background, both as a mother of a child with a life‐limiting illness and as a paediatric nurse, might influence the analysis and interpretation of the results. However, the researcher's genuine interest in other people's experiences and a thorough understanding of the background concepts supported the researcher in concluding the study.

### 6.2 Implications for Policy and Practice

According to the parents' experience, individualised family‐centred home‐based care not only improves the quality of life of children and families, but also reduces infections, complications and the number of hospital visits and in‐patient periods. In addition, it may possibly reduce the need for further psychosocial support for families and save costs to society. Parents are committed to the care of their child at home as much as possible if they are provided with individualised, family‐centred support throughout integrated (Cassidy et al. 2023) health and social services that are universal and publicly funded in Finland.

The experiences of FCC among the parents in this study represent a foundation for further exploration and conversation about how to prioritise and allocate finite healthcare resources, where to focus care improvement efforts and future research, and how to promote the use of digital opportunities in care, particularly in the home‐based setting for seriously ill children and their families (Rosenlund et al. 2023).

## 7 Conclusion

Family‐centred HBPC enables families to continue family life as normally as possible and allows all family members to be present at the bedside of the seriously ill child as much as they wish throughout the day. However, the main responsibility for the child's care often remains with the parents, regardless of whether there is a 24‐h care team at home or not, which may create an overwhelming burden for the families.

In future research, more attention should be focused on the assessment of the individual resources and needs of the parents and all family members in HBPC of children with life‐limiting illnesses to be able to offer family‐centred, appropriately tailored interventions with carefully integrated social and health services.

## Author Contributions

K.S., P.T., H.M., K.O. Critical contributions to study design. K.S. Substantial contributions to the conception and design, the acquisition, analysis and interpretation of the data. P.T., K.O. Supervision. K.S. Drafting the work and the manuscript. K.S., P.T., H.M., K.O. Critical revision, editing of the manuscript and final approval of the version to be published. K.S. Takes full responsibility for the integrity of the data and the accuracy of the data analysis.

## Conflicts of Interest

The authors declare no conflicts of interest.

## Supporting information


Data S1.

## Data Availability

Research data are not shared.
