# Supplementary material for: Parents' Experiences of Family‐Centred Care in Home‐Based Paediatric Care of Their Child With Life‐Limiting Illness: A Qualitative Descriptive Study
Source: J Adv Nurs. 2025 Mar 24;81(12):8780–93. doi: 10.1111/jan.16898 (PMC12623667; doi:10.1111/jan.16898)
Supplement: Supplementary file 1 — Data S1. [file JAN-81-8780-s001.docx › jan16898-sup-0002-TableS2.docx]

Supplementary table 2. Researchers’ characteristics.

| Author (gender) | Credentials,  Occupation at the time of the study, Country | Education on qualitative research | Experience in qualitative research |  |
| --- | --- | --- | --- | --- |
| Sari Karjula  (female) | RN,  Master in health management science at the University,  Finland. | She has completed formal qualitative research study modules at Master’s level. | She has conducted qualitative research studies. |  |
| Tarja Pölkki  (female) | PhD, RN,  Professor in nursing science at the University,  Finland. | She has completed formal qualitative research study modules at Master’s and PhD levels. | She has conducted qualitative research studies, taught qualitative research methods and written methodological articles related to qualitative content analysis. She has supervised Bachelor’s, Master’s and PhD theses which have used qualitative research methods. |  |
| Minna Hökkä  (female) | RN (Master’s degree), PhD,  Senior Advisor at the University of Applied Science, Finland. | She has completed formal qualitative research study modules at Master’s and PhD levels. | She has conducted qualitative research studies and taught qualitative research methods. She has supervised Bachelor’s and Master’s and PhD theses which have used qualitative research methods. |  |
| Outi Kanste  (female) | | PhD, RN,  Professor in health management science at the University,  Finland. | She has completed formal qualitative research study modules at Master’s and PhD levels. | She has conducted qualitative research studies, taught qualitative research methods and written methodological articles related to qualitative content analysis. She has supervised Bachelor’s, Master’s and PhD theses which have used qualitative research methods. |
